# Donor‐Dependent and Other Nondefined Factors Have Greater Influence on the Hepatic Phenotype Than the Starting Cell Type in Induced Pluripotent Stem Cell Derived Hepatocyte‐Like Cells

**DOI:** 10.1002/sctm.16-0029

**Published:** 2017-04-29

**Authors:** James A. Heslop, Richard Kia, Christopher S. Pridgeon, Rowena L. Sison‐Young, Triantafillos Liloglou, Mohamed Elmasry, Stephen W. Fenwick, John S. Mills, Neil R. Kitteringham, Chris E. Goldring, Bong K. Park

**Affiliations:** ^1^ MRC Centre for Drug Safety Science, Division of Molecular & Clinical Pharmacologythe Institute of Translational Medicine, the University of LiverpoolLiverpoolL69 3GEUnited Kingdom; ^2^ Department of Molecular and Clinical Cancer Medicinethe Institute of Translational Medicine, the University of LiverpoolLiverpoolL69 3GEUnited Kingdom; ^3^ University Hospital Aintree, Longmoor LaneLiverpoolL9 7ALUnited Kingdom; ^4^ AstraZeneca, Personalised Healthcare and Biomarkers, Alderley ParkCheshireSK10 4TGUnited Kingdom

**Keywords:** Primary human hepatocyte, Hepatocyte‐like cell, Epigenetic memory, Induced pluripotent stem cell, Donor‐dependent

## Abstract

Drug‐induced liver injury is the greatest cause of post‐marketing drug withdrawal; therefore, substantial resources are directed toward triaging potentially dangerous new compounds at all stages of drug development. One of the major factors preventing effective screening of new compounds is the lack of a predictive in vitro model of hepatotoxicity. Primary human hepatocytes offer a metabolically relevant model for which the molecular initiating events of hepatotoxicity can be examined; however, these cells vary greatly between donors and dedifferentiate rapidly in culture. Induced pluripotent stem cell (iPSC)‐derived hepatocyte‐like cells (HLCs) offer a reproducible, physiologically relevant and genotypically normal model cell; however, current differentiation protocols produce HLCs with a relatively immature phenotype. During the reprogramming of somatic cells, the epigenome undergoes dramatic changes; however, this “resetting” is a gradual process, resulting in an altered differentiation propensity, skewed toward the lineage of origin, particularly in early passage cultures. We, therefore, performed a comparison of human hepatocyte‐ and dermal fibroblast‐derived iPSCs, assessing the impact of epigenetic memory at all stages of HLC differentiation. These results provide the first isogenic assessment of the starting cell type in human iPSC‐derived HLCs. Despite a trend toward improvement in hepatic phenotype in albumin secretion and gene expression, few significant differences in hepatic differentiation capacity were found between hepatocyte and fibroblast‐derived iPSCs. We conclude that the donor and inter‐clonal differences have a greater influence on the hepatocyte phenotypic maturity than the starting cell type. Therefore, it is not necessary to use human hepatocytes for generating iPSC‐derived HLCs. Stem Cells Translational Medicine *2017;6:1321–1331*

**Significance Statement**

Current protocols for producing hepatocyte‐like cells (HLCs) do not produce fully mature hepatocytes. Previous reports have suggested an improved phenotype may be yielded by using the incomplete resetting of the epigenome during induced pluripotent stem cell (iPSC) generation. We report the first comparison of iPSCs derived from human isogenic hepatocytes and fibroblasts for producing HLCs. We find little significant difference between iPSC‐HLCs derived from hepatocytes and the easier to access and reprogram fibroblasts—an important finding for current and future studies which require patient specific (i.e., for transplantation) or disease/genotype specific phenotypes.

## Introduction

Drug‐induced liver injury (DILI) confers major clinical burden onto health care providers 1, 2 and is the most common cause of post‐marketing drug withdrawal 3, yet a method to accurately predict a toxic liability or model the mechanisms which causes DILI remains elusive. This is in part due to the lack of a translatable in vitro model which reliably triages potentially dangerous compounds during drug development or allows for mechanistic analyses. The gold standard for investigating DILI is the primary human hepatocyte (PHH) due to their metabolic competence; however, PHH dedifferentiate rapidly in culture and vary greatly between donors 4. Conversely, hepatic cell lines are scalable and readily available but only represent a single (abnormal) genotype and lack metabolic relevance 5. Therefore, an expandable, metabolically relevant, reproducible, population‐representative, and physiologically/genotypically normal source of cells would be an invaluable resource for investigating and predicting DILI.

One such resource which has the potential to address the role of hepatocytes in hepatotoxicity is induced pluripotent stem cells (iPSC)‐derived hepatocyte‐like cells (HLCs). Consequently, the generation of HLCs has been an area of intense research since the first differentiation protocols were described in 2004 by Lavon et al. 6. In the intervening years, incremental improvements have been made in both efficiency of differentiation and phenotype maturity. Despite these improvements, the hepatic phenotype still remains closer to a fetal or neonatal expression profile rather than an adult phenotype 7, 8, 9. This has led researchers to look elsewhere for improvements in the differentiation of stem cells toward HLCs, including the use of different extracellular substrates 10, three‐dimensional culture systems 11, small molecules 12, and products of the microbiome 13.

One further area of interest has been to investigate the cell of origin. Previous work has shown that during the reprogramming process, the hypermethylation of the genes associated with the somatic cell phenotype occurs at a relatively late stage of induced pluripotency 14. Consequently, iPSCs maintain a memory of the cell from which they were derived 15. This phenomenon, termed epigenetic memory, was first described by two separate studies in 2010 16, 17 demonstrating that the starting cell type can influence the differentiation propensity of the iPSC toward the lineage of origin. This was subsequently found to be true of other cell types, such as pancreatic β cells, which demonstrated increased PDX1 and insulin gene expression in spontaneously differentiated iPSCs‐derived from β cells when compared to non‐β cell‐derived iPSCs and embryonic stem cells (ESCs) 18.

Epigenetic memory has also been investigated using hepatocytes (Supporting Information Table S1); however, no study has provided definitive proof of whether the starting cell type significantly impacts upon the hepatic differentiation capacity of iPSCs. Genotype‐controlled experiments in mice have shown a transient advantage in HLC gene expression of albumin and CK18 in hepatic‐derived iPSCs compared to mouse embryonic fibroblast (MEF)‐derived iPSCs 19. However, this advantage was greater when iPSCs were derived from hepatoblasts than mature hepatocytes and dissipated with time in culture. Liu et al. 20, published the first human comparison in 2011, demonstrating no significant advantage in hepatic phenotype when PHH‐derived iPSCs were compared to iPSCs derived from other cell types derived from different donors, as measured by albumin secretion, Cytochrome P450 (CYP)3A activity assays; however, a similar but distinct global epigenetic profile was reported 20.

More recently, Takayama et al. 21 corroborated the report from Lee et al. 19 in human cells, demonstrating a small but significant advantage when hepatocytes were compared to other cell types; however, the cells were sourced from different donors and the advantage was again found to dissipate with time in culture. The importance of donor‐dependent genetic differences was further highlighted by Kajiwara et al. 22, who showed that donor differences were the largest determinant of HLC quality, rather than the cell of origin, although this study did not use PHH‐derived iPSCs.

Therefore, the investigation of epigenetic memory in HLCs can only be truly assessed using a comparison of human genotype‐controlled iPSCs derived from hepatocytes and non‐hepatocytes isolated from the same donor. Herein, we describe the comparison of isogenic PHH‐ and dermal fibroblast derived iPSCs and the hepatic differentiation potential of these cells.

## Methods

### PHH and Dermal Fibroblast Isolation and Culture

Liver and skin resections were received as surgical waste (Aintree hospital, Liverpool, UK) with full patient consent and ethical approval from the relevant institutional review boards (National Research Ethics Service REC ref: 11/NW/0327). PHHs were isolated using a modified version of the previously described 2‐step collagenase method as previously described 23.

Human dermal fibroblasts (HDFs) were isolated using a previously published protocol 24. Skin biopsies were cut using scalpel and forceps into approximately 1 cm^2^ pieces and placed into a six‐well plate (3 pieces/well). Two drops of Dulbecco's modified Eagle's medium (DMEM; Sigma Aldrich, St. Louis, MO, www.sigmaaldrich.com) supplemented with 10% (v/v) fetal bovine serum (FBS; Life technologies, Carlsbad, CA, www.thermofisher.com/), 2 mM l‐glutamine (Sigma‐Aldrich) and 1% (v/v) penicillin/streptomycin (Sigma‐Aldrich; HDF media) were placed on top of each segment and left to attach overnight. Drops of HDF media were replaced daily to keep the pieces submerged. After 7 days, 1 ml of media was added/well and changed every 48 hours. After 2 weeks HDF outgrowths were observable. Once confluent, HDFs were detached with 0.05% trypsin (Sigma Aldrich) and expanded for reprogramming and cryopreservation.

### Sendai Virus Reprogramming of PHH and HDFs

PHH were plated on ESC‐qualified Matrigel (Corning, Corning, NY, www.corning.com) at a density of 1 × 10^5^ to 5 × 10^5^ cells/well in a six‐well plate. Cells were cultured in Williams E medium supplemented with 2.5% (v/v) FBS (Life technologies), 50 ng/ml hepatocyte growth factor (HGF; Promokine, Heidelberg, Germany, www.promokine.info), 50 ng/ml epidermal growth factor (EGF, Life technologies), 15 mM 4‐(2‐hydroxyethyl)‐1‐piperazineethanesulfonic acid (HEPES) buffer (Sigma‐Aldrich), 100 nM dexamethasone (Sigma‐Aldrich), 0.2% (v/v) insulin‐transferrin‐selenium (Life technologies) and 0.5% (v/v) penicillin/streptomycin (PHH reprogramming media). Following 3 days of culture, Sendai viruses containing Oct4, Sox2, Klf4, and c‐Myc transcription factors (Life technologies) were added at multiplicities of infection (MOI) 3–10. After 24 hours, media was replaced with non‐virus containing PHH reprogramming media and culture continued for a further 2 days. Subsequently, cells were then cultured in Essential 6 media (Life technologies) containing basic fibroblast growth factor (100 ng/ml; Life technologies) and 0.5% (v/v) penicillin/streptomycin for 25–30 days, replacing the media every 24 hours.

HDFs were plated in a six‐well plate at 5 × 10^6^ cells/well or 3 × 10^5^ cells/well, respectively. After 48 hours of culture, cells were transduced with Sendai viruses containing Oct4, Sox2, Klf4, and c‐Myc at MOI 3 or 5. After 24 hours, media was replaced and changed every 48 hours for 7 days. On day 7 of reprogramming, cells were trypsinized (0.05% trypsin) and replated onto 10 cm^2^ dishes coated with MEFs (Globalstem, Rockville, MD, www.mti-globalstem.com) or six well plates coated with Matrigel. Cells were plated in HDF media which was replaced with either DMEM/F12 media supplemented with 20% (v/v) KnockOut serum replacement media, ×1 nonessential amino acids, 50 µM 2‐mercaptoethanol (Life technologies) and 0.5% (v/v) penicillin/streptomycin on MEF cultures or Essential 6 media supplemented 100 ng/ml basic‐Fibroblast growth factor (FGF) (Life technologies) for Matrigel cultures. Media was changed daily and colonies formed from day 21 onward. iPSCs were maintained on either MEF coated plates in DMEM/F12 media supplemented with 20% (v/v) KnockOut serum replacement media, ×1 nonessential amino acids, 50 µM 2‐mercaptoethanol and 0.5% (v/v) penicillin/streptomycin or on Matrigel in Essential 8 medium. All early passage comparison assays were performed using <p10 (donors 1 and 2) or <p15 (donor 3) iPSCs. Characterization was performed using iPSCs >p15 and late passage analysis was conducted using iPSCs >p30.

### Differentiation to HLCs

Cells were transferred to Matrigel culture 1:1 from MEF culture and cultured in Essential 8 medium. Upon confluence, cells were washed once with Dulbecco's phosphate buffered saline (DPBS) and disassociated with Accutase at 37°C for ∼5 minutes. Cells were then centrifuged at 200*g* for 5 minutes and resuspended in Roswell Park Memorial Institute (RPMI) media supplemented with ×1 B27 and 10 µM Rho‐associated protein kinase (ROCK) inhibitor (Merck Millipore, Billerica, MA, www.merckmillipore.com). Cells were then counted and plated at 1.5 × 10^5^ cells/cm^2^ on Matrigel coated 24 well plates in RPMI media (Life technologies) supplemented with ×1 B27 (Life technologies), 0.5% (v/v) penicillin/streptomycin, 10 µM Roswell ROCK inhibitor (Merck Millipore), 100 ng/ml Activin A and 50 ng/ml Wnt3a (R&D Systems, Minneapolis, MN, www.rndsystems.com). Following overnight plating, cell media was replaced daily with RPMI media containing ×1 B27, 0.5% (v/v) penicillin/streptomycin, 100 ng/ml Activin A and 50 ng/ml Wnt3a. After 3 days, Wnt3a was omitted from the media for further 2 days. At day 5, media was replaced with KnockOut DMEM media containing 20% (v/v) KnockOut serum, 1 mM l‐glutamine, 0.5% (v/v) penicillin/streptomycin, ×1 nonessential amino acids, 100 µM 2‐mercaptoethanol and 1% (v/v) dimethyl sulfoxide (DMSO). Media was changed every 48 hours for 7 days. At day 12, media was replaced with HepatoZyme culture media (Life Technologies) supplemented with 2 mM l‐glutamine, 0.5% (v/v) penicillin/streptomycin, 20 ng/ml HGF, 20 ng/ml Oncostatin M (OSM) (Promokine), and 100 nM dexamethasone. At day 22, cells were lysed for HLC comparisons. Samples were also taken at definitive endoderm (day 5) and hepatic endoderm (H.E; day 12) stages.

### Spontaneous Differentiation Assays

Cells were disassociated using gentle cell disassociation reagent (Stem Cell Technologies; Vancouver, British Columbia, Canada, www.stemcell.com) and scraped in to DMEM/F12 media supplemented with 20% (v/v) KnockOut serum, ×1 nonessential amino acids, 100 µM 2‐mercaptoethanol, 0.5% (v/v) penicillin/streptomycin and 10 µM ROCK inhibitor. MEFs were removed by gravitational separation and cells plated in 12‐well non‐tissue culture treated plates (Corning) in triplicate (1:1 ratio). Media was changed every 48 hours without ROCK inhibitor. For gene expression comparisons, cells were cultured for 16 days before lysing in QIAzol (QIAgen, QIAgen, Manchester, UK; www.qiagen.com). For characterization experiments, cells were cultured for 7 days, before transfer to attachment factor‐coated 48 well tissue‐culture treated plates for reattachment. Cells were cultured for a further 7 days, before fixing with 4% (v/v) PFA for immunofluorescence assessment.

### Pyrosequencing

DNA was extracted using the QIAamp DNA mini kit according to the manufacturer's instructions (QIAgen). DNA/sample (250 ng) was then bisulfite converted using the EZ DNA Methylation‐Gold kit (Zymo, Irvine, CA, www.zymoresearch.com) according to the manufacturer's protocol. Genes bearing CpG islands within their promoter region were ascertained using the NCBI gene information and the online tool CpG island searcher (http://cpgislands.usc.edu/). Polymerase chain reaction (PCR) and pyrosequencing primers (sequencing, biotinylated and non‐biotinylated, Supporting Information Table S2) were designed using the Pyromark Assay Design 2.0 software (QIAgen, www.eurofins.com) and purchased from Eurofins (Eurofins, Luxembourg). PCR products were generated from the bisulfite‐converted samples for all primer sets using optimized conditions. Single‐strand pyrosequencing templates were generated from PCR product following binding to streptavidin beads and subsequent washes with 70% ethanol, 0.2 M NaOH, and 10 mM Tris‐Acetate pH 7.5. Pyrosequencing was undertaken on a PyroMark Q96 ID instrument (QIAgen).

### Immunofluorescence

Cells were fixed with 4% (w/v) paraformaldehyde (Sigma Aldrich) for 15 minutes and subsequently washed three times with DPBS buffered with MgCl_2_ and CaCl_2_ (DPBS+; Life technologies). Fixed cells were blocked with DPBS+ supplemented with 10% (v/v) donkey serum (Sigma Aldrich) and 0.01% (v/v) Triton X‐100 (Sigma Aldrich) for 30 minutes. Primary antibodies were diluted (Supporting Information Table S3) in DPBS+ containing 1% (v/v) donkey serum 0.01% (v/v) Triton X‐100 and incubated overnight at 4°C. Cells were washed three times with DPBS+ and alexafluor secondary antibody diluted 1:750 in DPBS+ supplemented with 1% (v/v) donkey serum with 0.01% (v/v) Triton X‐100 added and incubated at room temperature for 2 hours. Cells were washed three times and Hoechst stain (1 mg/ml; Sigma Aldrich) added at a 1:7,500 dilution in DPBS+ for 10 minutes. Cells were washed three times and imaged using the Axio Observer Z1 fluorescence microscope with the AxioCam MR digital camera. Images were processed using Zen lite software (Carl Zeiss, Oberkochen, Germany; www.zeiss.com).

For albumin staining, ×10 blocking buffer (Abcam, Cambridge, UK, www.abcam.com) was used to replace donkey serum. Staining with Tra‐1‐60 and SSEA‐4 conjugated antibodies was performed without Triton X‐100 and with a single 90‐minute incubation at room temperature following blocking with 10% (v/v) donkey serum in DPBS+.

### qRT‐PCR Analysis of Gene Expression

Primers for use in quantitative reverse transcription (qRT)‐PCR gene expression studies were designed using the NCBI Primer BLAST tool (http://www.ncbi.nlm.nih.gov/tools/primer-blast/). Primer quality was assessed using OligoCalc and purchased from Eurofins (Supporting Information Table S4). Samples were collected in QIAzol (QIAgen), and extracted using the miRNeasy extraction kit according to the manufacturer's protocol (QIAgen) Extracted RNA content was measured using the NanoDrop spectrophotometer (Thermo Fisher Scientific). RNA was reverse transcribed to complementary DNA (cDNA) using the ImProm‐II reverse transcription kit (Promega).

Due to low RNA yield following extraction, whole transcriptome amplification was performed on selected samples using the QuantiTect Whole Transcriptome Kit (Qiagen), following the manufacturer's protocol. Briefly, 100 ng of starting RNA from each sample was mixed with reverse transcriptase kit component and incubated at 37°C for 30 minutes and 95°C for 5 minutes. Ligation mix was then added to each sample and incubated for 2 hours at 22°C. Finally, the amplification mix was added and incubated for 8 hours at 30°C and 5 minutes at 95°C. Temperature controlled steps were carried out using the GeneAmp PCR system 9700 thermal cycler system (Applied Biosystems, Waltham, MA, www.thermofisher.com) and amplification confirmed using the NanoDrop spectrophotometer.

Gene expression analysis was performed using the SYBRGreen JumpStart Taq ReadyMix (Sigma Aldrich). Briefly, 10–100 µg of cDNA was amplified by qRT‐PCR as follows: 95°C for 10 minutes, then 40 cycles for 95°C 15 seconds, and 60°C for 60 seconds using the ViiA7 qRT‐PCR machine (Applied Biosystems). Results were calculated using the C_T_ values generated, normalized against glyceraldehyde 3‐phosphate dehydrogenase (GAPDH) and succinate dehydrogenase 25 and calculated relative to a calibrator sample (e.g., PHH) using 2^−ΔΔCT^ method 26. Sendai virus expression analysis of iPSCs was conducted using the TaqMan iPSC Sendai Detection Kit (Life Technologies) according the manufacturer's instructions. Results were normalized using GAPDH and calculated relative to the reprogramming plate using 2^−ΔΔCT^ method.

### Metabolism Studies

Cells were incubated for 15 minutes at 37°C (5% CO_2_) in standard culture media with a final substrate cocktail concentration of 1 mM testosterone (CYP3A4) and 0.25 mM dextromethorphan (CYP2D6) (Sigma Aldrich) in MeOH or H_2_O, respectively (Thermo Fisher Scientific). Phenacetin (0.5 µM; Sigma Aldrich) in 100% MeOH was then added to the incubation media (1:1 v/v) as a stop solution and an internal standard for Liquid chromatography with tandem mass spectrometry (LC‐MS‐MS) analysis. The media containing the respective metabolites, 6β‐OH‐testosterone and dextrorphan, was then filtered using 96‐well filter plates (Merck Millipore) and analyzed by LC‐MS‐MS. Results were normalized by protein content of the well following quantification by Bradford assay.

### Albumin ELISA

The albumin concentration was measured by enzyme‐linked immunosorbent assay (ELISA) following the manufacturer's protocol (Bethyl Laboratories, Montgomery, TX) protocol. Briefly, 96‐well high‐affinity binding plates (Thermo Fisher Scientific) were coated with coating antibody in coating buffer for 1 hour and subsequently blocked with 1% (w/v) bovine serum albumin (Sigma Aldrich) for 30 minutes. Cell supernatants were added to wells at appropriate dilutions and incubated at room temperature for 1 hour. Horseradish peroxidase‐conjugated detection antibody was then added to each well at a 1:50,000 concentration. Following a 1 hour incubation, 3,3′,5,5′‐Tetramethylbenzidine substrate solution was added, incubated for 20 minutes and the reaction stopped with 0.18 M sulfuric acid (Thermo Fisher Scientific). Plates were read at 450 nm using a MRX^e^ Revelation plate reader (Dynex Technologies, Chantilly, VA, www.dynexproducts.com).

## Results

### Generation of iPSCs Derived From PHH and HDFs of Multiple Donors

To assess the differences between hepatocyte‐like cells (HLCs) differentiated from PHH‐ and HDF‐derived iPSCs, a reprogramming technique capable of inducing pluripotency in both cell types was required. Reprogramming of PHH has been reported as relatively inefficient compared to more traditionally used cell types, such as HDFs 27. Therefore, a technique capable of reprogramming PHH was first optimized.

PHH are known not to respond well to trypsinization and replating, therefore, it was hypothesized that developing a protocol which did not require replating would be the most successful route. Furthermore, given that the cell cycle is a vital component of reprogramming 28, the media composition described by Liu et al. 29, which contains high levels of known mitogenic factors HGF and EGF, was used (Supporting Information Fig. S1a).

Using these conditions, PHH were transduced with the OSKM‐containing Sendai viruses at MOI 5 and 10 and examined during the reprogramming period for colony formation (Supporting Information Fig. S2a). At day 20, the first colonies were noted with similar, but incomplete iPSC‐like morphology. These colonies appeared to be heterogeneous and underwent growth arrest during the culture period (Supporting Information Fig. S2a). At day 24, one colony was noted which exhibited the hallmark features of an iPSC colony (Supporting Information Fig. S2b). After tracking the colony over several days, the colony remained homogenous and continued to expand. This colony was manually picked for expansion and fully characterized.

**Figure 1:**
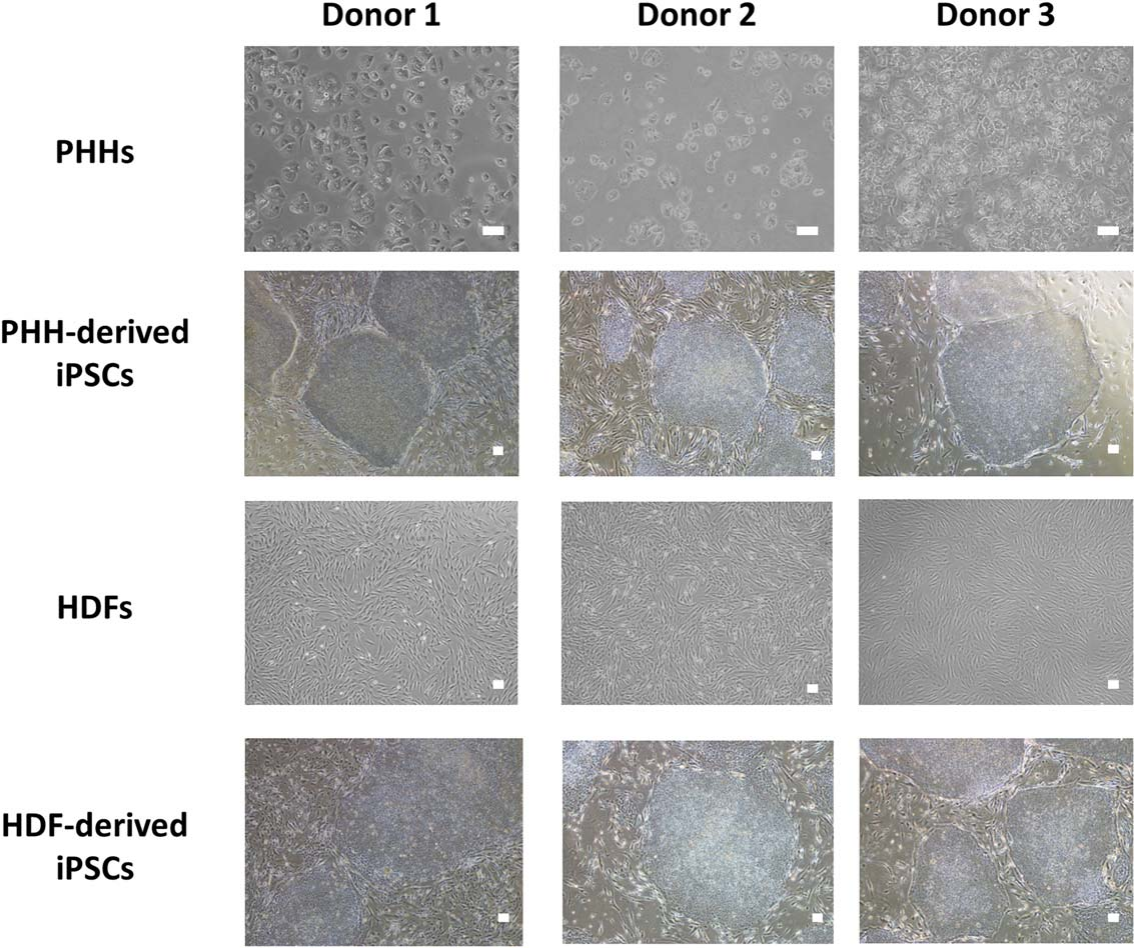
Isolation and reprogramming of somatic cells. The morphology of PHH and HDFs from each donor prior to reprogramming and selected examples of the iPSCs which were generated from each of these starting cell types/donors. PHH: Magnification: ×100, scale bar: 100 µm; iPSCs and HDFs: magnification ×40, scale bar: 100 µm. Abbreviations: HDF, human dermal fibroblast; iPSC, induced pluripotent stem cell; PHH, primary human hepatocyte.

Using this protocol, iPSCs were successfully generated from hepatocyte cultures from three separate donors (Fig. 1; Supporting Information Table S5). Subsequent to successful PHH‐reprograming, the dermal fibroblasts of the corresponding donors were also reprogrammed using a well‐established dermal fibroblast protocol (Supporting Information Fig. S1b). Pluripotency was assessed using a panel of ESC‐enriched markers: Oct4, Sox2, Nanog, Tra‐1‐60, and SSEA‐4 (Supporting Information Fig. S3). All lines examined demonstrated expression of each of the markers, indicating that they had established a pluripotent expression profile.

The functional differentiation capacity of these lines was also assessed using embryoid body (EB) assays. This assay encourages cellular differentiation and, if truly pluripotent, should result in differentiation toward cell types from all three germ layers: ectoderm, mesoderm, and endoderm. The presence of each of these germ layers within the differentiated cell population was investigated by immunofluorescence, using the markers TUJ1 (Neuron‐specific class III β‐tubulin; ectoderm), alpha smooth muscle actin (mesoderm), and alpha‐fetoprotein (AFP; endoderm) (Supporting Information Fig. S4). All of the iPSC lines generated stained successfully for these markers, indicating that all selected lines were functionally pluripotent.

### Donor‐Dependent Differences in PHH‐ and HDF‐Derived iPSCs Gene Expression Profile

**Figure 2:**
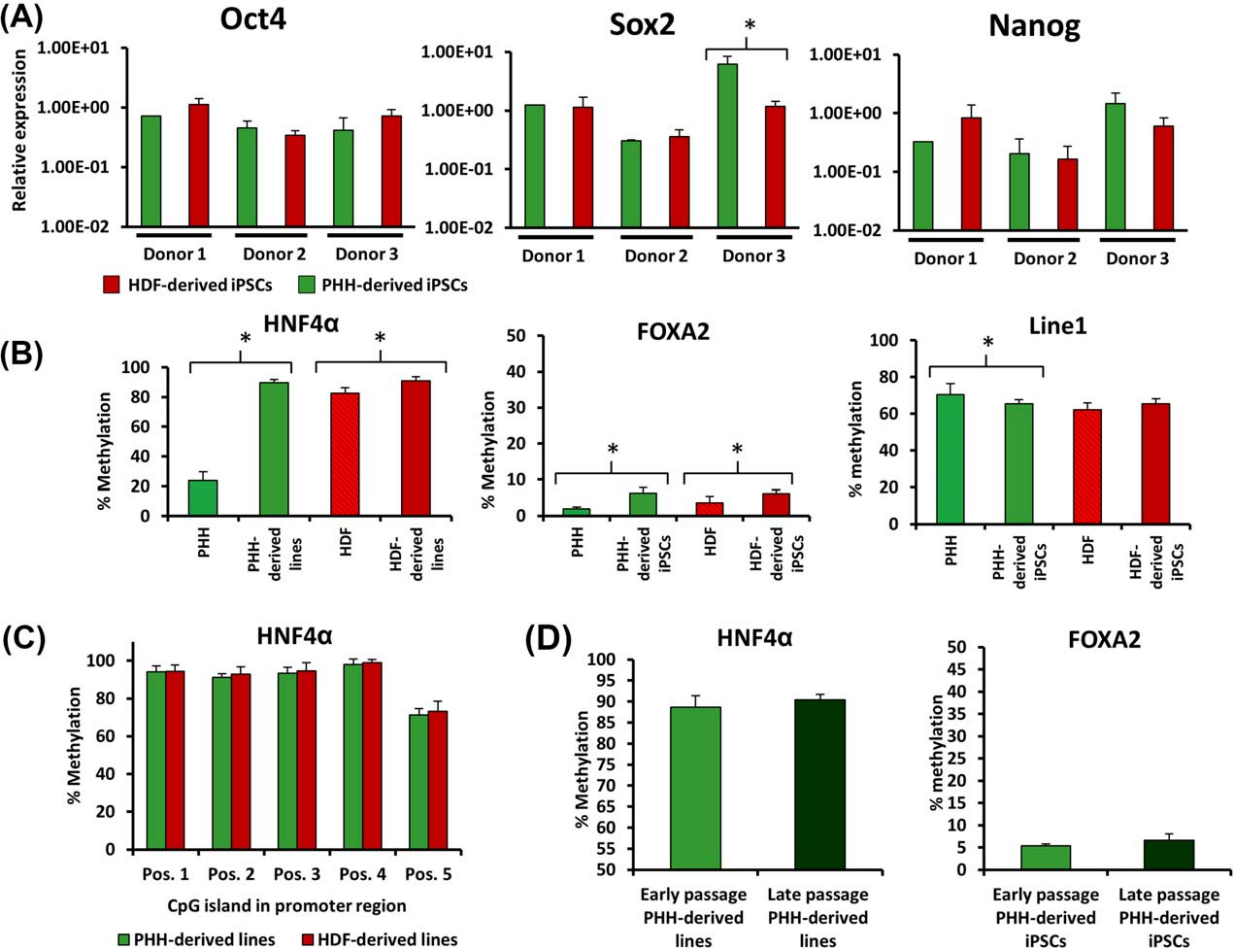
Comparison of iPSC gene expression by quantitative reverse transcription‐polymerase chain reaction (qRT‐PCR) and methylation status by pyrosequencing. **(A)**: Nanog, Sox2, and Oct4 gene expression determined by qRT‐PCR and presented as 2^−ΔΔCT^ relative to hESC comparator and normalized with glyceraldehyde 3‐phosphate dehydrogenase. (*) Denotes *p* > .05 unpaired T test. **(B)**: The changes in methylation status of HNF4α, FOXA2, and LINE1 in PHH (48 hours culture) and HDFs pre‐ and post‐reprogramming. (*) Denotes *p* > .05 one‐way analysis of variance (ANOVA). **(C)**: The difference in HNF4α methylation status of the PHH‐ and HDF‐derived iPSCs across the 5 CpG sites located in the analyzed sequence. **(D)**: The methylation status of HNF4α and FOXA2 which occur following repeat passaging of the cells during standard culture procedures. Abbreviations: HDF, human dermal fibroblast; hESC, human embryonic stem cell; iPSC, induced pluripotent stem cell; PHH, primary human hepatocyte.

To fully compare the iPSCs generated from PHH and HDFs, comparisons were required at all stages of culture and differentiation. Therefore, the expression of important pluripotency‐associated genes at the iPSC‐stage was compared. Across these markers, Oct4 and Nanog showed similar levels of expression between both cell types of origin. Sox2 expression was significantly greater in the PHH‐derived lines for donor 3; however, this was not true of donors 1 and 2 (Fig. 2A). Furthermore, analysis of Wnt3, which has been proposed as an iPSC marker of definitive endoderm potential (i.e., high Wnt3 expression correlates with high purity definitive endoderm [D.E] formation) 30, again showed no significant differences between PHH and HDF‐derived iPSCs; however, a donor‐dependent difference was observed (Supporting Information Fig. S5).

### HNF4α Promoter Analysis in PHH‐ and HDF‐Derived iPSCs Demonstrates a Small but Consistent Cell‐Type Specific Difference in Methylation Which Decreases with Time in Culture

As the inherent memory of iPSCs is reported to be a consequence of incomplete epigenetic reprogramming, the potential differences seen between PHH‐ and HDF‐derived lines at the iPSC stage were investigated. In this context, we assessed the methylation status of key hepatic genes: HNF4α and FOXA2, since their epigenetic status would likely influence the differentiation status of the HLCs. During the process of reprogramming, the methylation of HNF4α and FOXA2 promoter regions in both PHH and HDFs was significantly increased to similar levels between PHH and HDF‐derived clones (Fig. 2B). We assessed global methylation using an assay for Long interspersed nuclear elements retrotransposable element 1, which is repeated throughout the genome and, therefore, often used as a surrogate marker of whole genome methylation status 31. Our results indicated very similar global methylation indexes between PHH‐ and HDF‐derived iPSCs (65.35% and 65.43%, respectively; Fig. 2B).

Notwithstanding the global methylation similarity, across the five CpG sites examined within the HNF4α promoter region, each site was consistently ∼1% less methylated in the PHH‐derived lines when compared to the HDF‐derived lines (Fig. 2C). Across all lines and CpG sites investigated, 1.22% less methylation was observed in PHH‐derived samples; however, this trend was found to decrease with time in culture, with a difference of 0.42% methylation between late passage PHH‐derived and HDF‐derived iPSCs (Fig. 2D). No conserved methylation differences were found between PHH and HDF‐derived iPSCs in the FOXA2 promoter; however, FOXA2 methylation was found to increase (1.22%) between early and late passage iPSCs.

### Spontaneous Differentiation of PHH‐ and HDF‐Derived iPSCs Reveals the Greatest Discriminator Between the iPSCs Is the Donor, Rather than the Starting Cell Type

To investigate if the cell‐type of origin influences the inherent differentiation propensity of iPSCs, the hepatic/endoderm‐enriched gene expression of EBs was compared. Using a previously published method for comparing iPSCs of different origins 18, EBs were generated and cultured in suspension for 16 days. The expression of known endoderm and hepatic markers was then compared.

**Figure 3:**
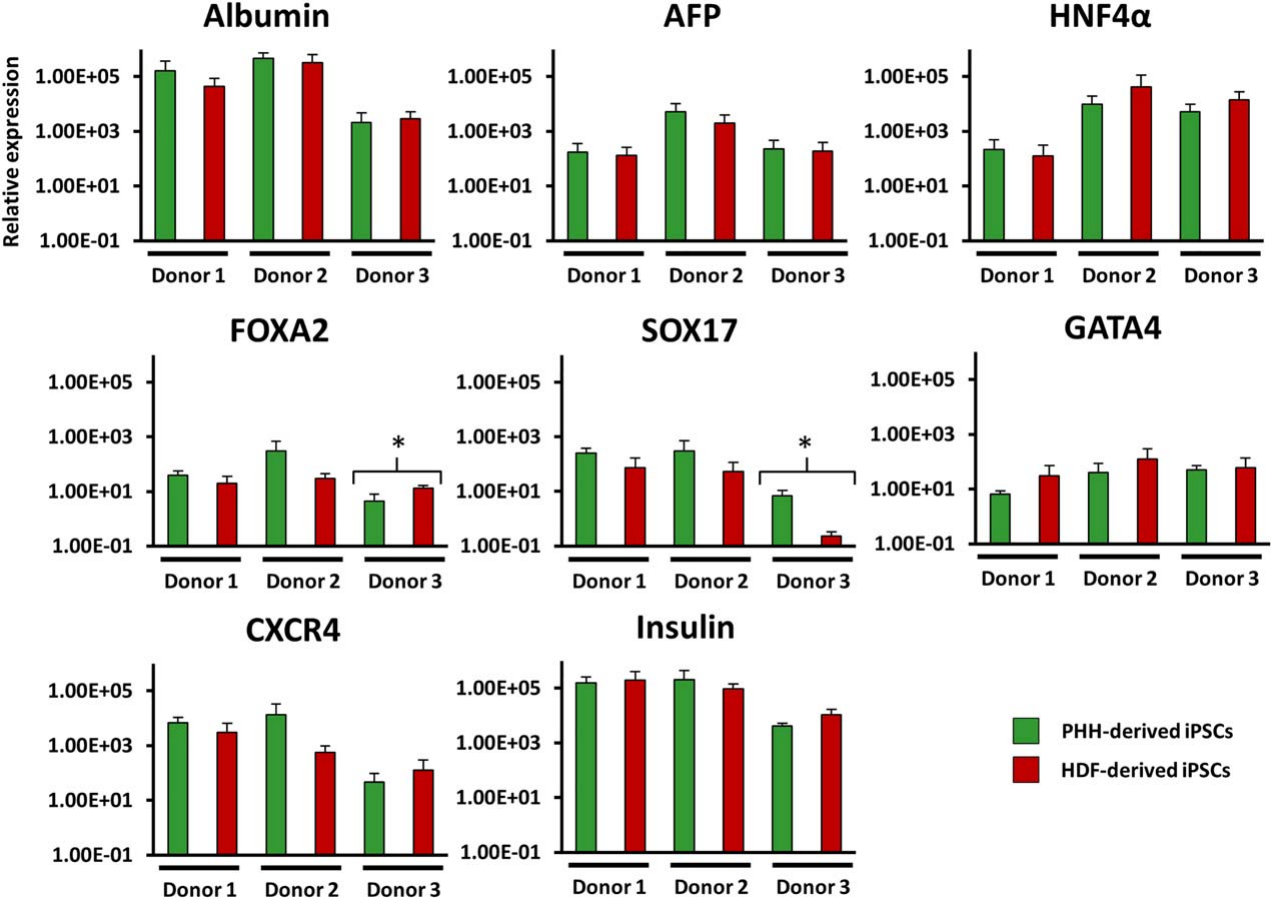
Comparison of endoderm and hepatic associated gene expression in PHH‐ and HDF‐derived embryoid bodies using quantitative reverse transcription‐polymerase chain reaction (qRT‐PCR). All genes shown as 2^−ΔΔCT^ relative to a hESC‐derived embryoid body comparator and normalized to glyceraldehyde 3‐phosphate dehydrogenase and succinate dehydrogenase. Error bars represent SD between the means of each PHH‐/HDF‐derived induced pluripotent stem cell line which were derived from three individual differentiation cultures. Each sample tested by qRT‐PCR was loaded in duplicate. (*) Denotes *p* > .05 unpaired T test. Abbreviations: AFP, alpha‐fetoprotein; HDF, human dermal fibroblast; PHH, primary human hepatocyte.

The results presented in Figure 3 suggested a very slight trend toward the enhancement of hepatic‐associated gene expression in PHH‐derived EBs in five of the genes tested, that is, Albumin, AFP, SOX17, FOXA2, and CXCR4. However, GATA4 and HNF4α showed a trend toward higher expression in the HDF‐derived clones. Despite these trends, only Sox17 showed significantly greater expression in PHH‐derived iPSCs, although this difference was only observed in one of the three donors. Taken together there was very little difference in the spontaneous differentiation propensity of PHH and HDF‐derived iPSCs across the investigated hepatic and endodermal‐associated genes. Indeed, the greatest determinant of gene expression appeared to be the donor, rather than the starting cell type.

### Donor‐ and Clonal‐Dependent Variation Are a Greater Influence on the Gene Expression of HLCs than the Starting Cell Type

The differentiation of iPSCs toward HLCs was achieved using a protocol adapted from previous publications 22, 32 (Supporting Information Fig. S6a) and successful differentiation was confirmed by immunofluorescence at each stage: D.E (Sox17), H.E (HNF4α), and HLCs (Albumin) (Supporting Information Fig. S6b).

To fully evaluate the differentiation procedure and to assess any inherent advantage as a result of the different starting cell types, we compared the gene expression at each stage of differentiation. Supporting Information Figure S7a demonstrates a comparison of several key markers of definitive endoderm. The results followed a similar pattern to previous analysis, demonstrating no significant differences between starting cell types when the gene expression of FOXA2, SOX17, and GATA4 was compared. Interestingly, both GATA4 and FOXA2, showed a larger dependence on the donor of origin than starting cell type.

Similar analysis was also undertaken for the hepatic endoderm stage of differentiation, using AFP and HNF4α as stage‐specific markers. Despite a trend toward enhanced levels of both markers in the PHH‐derived iPSCs, due to inter‐clonal variation, only HNF4α in donor 2 demonstrated significantly enhanced gene expression in PHH‐derived hepatic endoderm (Supporting Information Fig. S7b).

**Figure 4:**
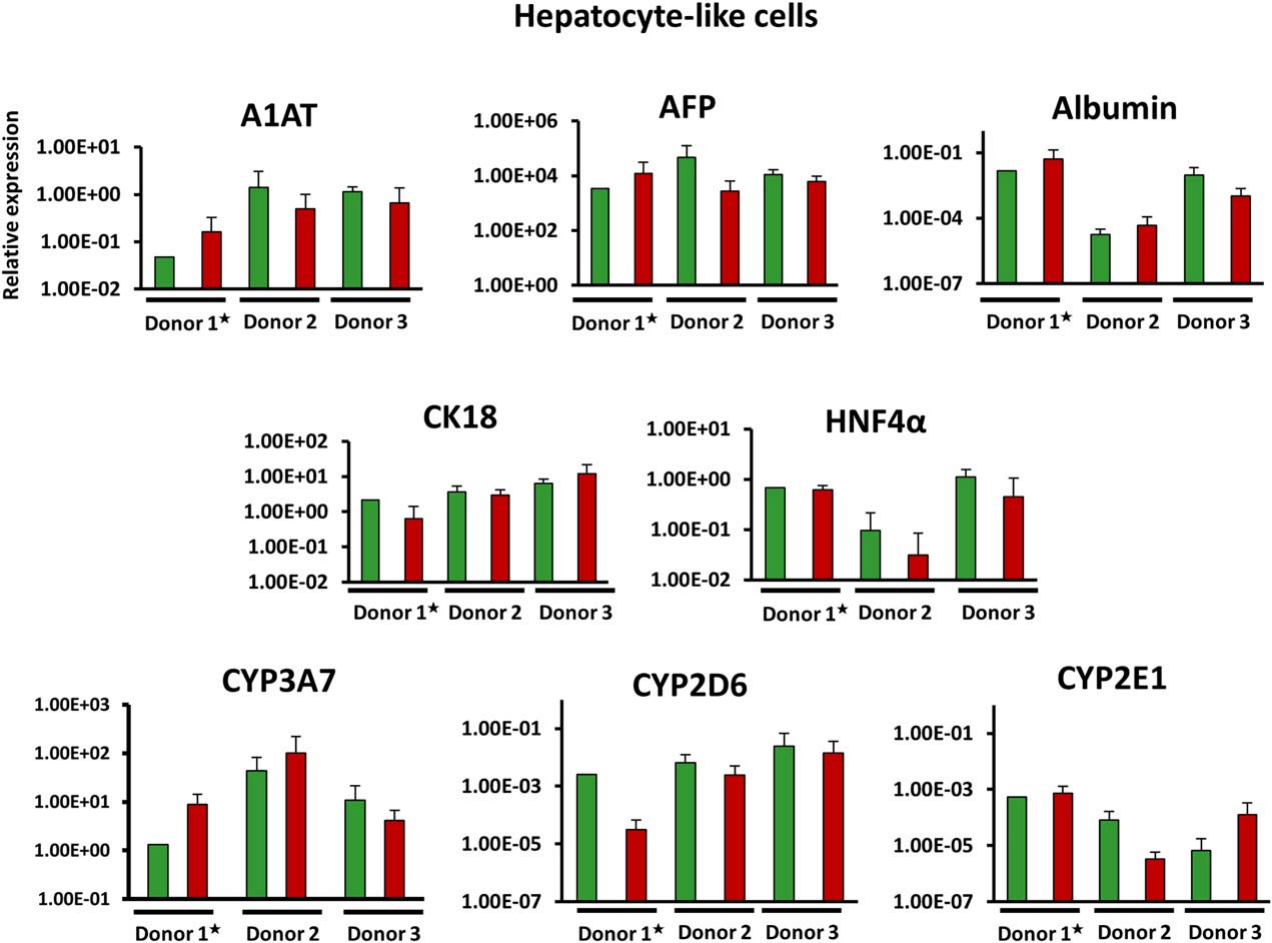
Comparison of hepatocyte‐like cell gene expression measured by quantitative reverse transcription‐polymerase chain reaction (qRT‐PCR). Gene expression of key hepatic genes obtained by q‐PCR analysis. Results normalized using glyceraldehyde 3‐phosphate dehydrogenase and succinate dehydrogenase and displayed as 2^−ΔΔCT^ relative to primary human hepatocyte (PHH) of the corresponding donor. ^**★**^N.B. Donor 1 shown relative to isogenic plated PHH; donor 2 and 3 shown relative to non‐plated isogenic PHH. Error bars represent SD between the means of each PHH‐/human dermal fibroblast‐derived induced pluripotent stem cell line which were derived from three individual differentiation cultures. Each sample tested by qRT‐PCR was loaded in duplicate. (*) Denotes *p* > .05 unpaired T test. Abbreviation: AFP, alpha‐fetoprotein.

**Figure 5:**
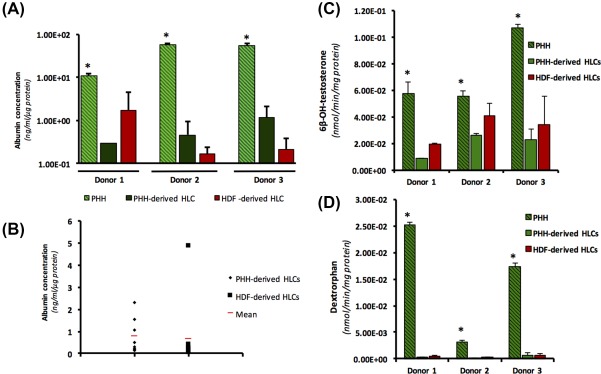
Functional comparison of hepatocyte‐like cells. **(A)**: Albumin secretion in PHH and PHH‐ and HDF‐derived HLCs at the final stage of differentiation measured by ELISA and normalized using total protein content of corresponding well. (*) Denotes *p* > .001 one‐way ANOVA. Error bars: SD. **(B)**: Albumin secretion comparison of PHH and HDF‐derived HLCs from all three donors *p > .05 unpaired T test. **(C)** and **(D)**: CYP activity as detected using LC‐MS‐MS method analyzing the turnover of probe substrates and quantification of metabolites for (C) 6β‐OH‐testosterone (CYP3A) and (D) Dextrorphan (CYP2D6). (*) Denotes *p* > .05 one‐way ANOVA. Error bars: SD. Abbreviations: HDF, human dermal fibroblast; HLC, hepatocyte‐like cell; PHH, primary human hepatocyte.

Final stage HLCs were then compared using a panel of mature and immature hepatic markers alongside functional assays with the individual donor's PHH used as the gold standard comparator (Figs. 4, 5 and Supporting Information Fig. S8).

Gene expression analysis of these markers demonstrated no significant differences between the lines generated from different cell types in any of the donors. Despite this, some genes did show a trend toward improved gene expression in the PHH‐derived clones; for example, in all three donors, greater expression of HNF4α and CYP2D6 is found in PHH‐derived HLCs when compared to the corresponding donor's HDF‐derived HLCs. Conversely, the other markers lack conformity, with the greater expression of the marker gene differing between PHH and HDF‐derived HLCs across the assessed donors (Fig. 4C).

### Functional Comparisons Show No Significant Difference Between PHH‐ and HDF‐Derived HLCs

To assess the functional capacity of the HLCs, albumin secretion was compared at day 22 of differentiation. The data demonstrated a nonsignificant trend pointing toward PHH‐derived clones having slightly higher, but nonsignificantly different, albumin secretion in two of the three donors (Fig. 5A). When examined together, the difference between PHH‐ and HDF‐derived HLC albumin secretion was not statistically significant (Fig. 5B).

CYP3A and CYP2D6 activity was also assessed using probe substrates testosterone and dextromethorphan, respectively (Fig. 5C, 5D). Again, no significant differences were found between the HLCs derived from the different starting cell types.

## Discussion

In this study, we have investigated the hepatocyte differentiation potential of PHH‐ and HDF‐derived iPSCs derived from the same donor in a multifaceted panel of assays, to give a thorough comparison of the hepatic phenotype.

Our main hypothesis, that the starting cell type influences the differentiation capacity of the cell, was driven by the reported epigenetic memory which remains from the somatic cell following reprogramming 16, 17. Our results suggested a small but statistically nonsignificant epigenetic memory may be found in the developmentally important liver‐enriched transcription factor HNF4α promoter region in reprogrammed cells derived from human hepatocytes 33, 34. This is in contrast to 25% difference in the methylation of the PDX1 gene promoter region reported between β‐pancreatic cell‐derived iPSC and non‐β‐pancreatic cell‐derived iPSCs 18. Throughout the analysis, a general trend toward a slight, nonsignificant advantage, to starting with PHHs, when compared to dermal fibroblasts, was also noted. This was most evident with HNF4α expression at the hepatic endoderm stage of differentiation. Therefore, the slightly enhanced phenotype in PHH‐derived iPSCs at the HLC stage may be driven by the small differences in the methylation patterns of HNF4α reported in this study and other hepatic‐associated genes. Moreover, the lack of a clear enhancement in cell differentiation, as described in pancreatic β cell‐derived iPSCs 18, may be a consequence of the relatively small differences in the epigenetic profile of PHH‐ and HDF‐derived iPSCs. Our findings, that the noted differences in HNF4α methylation decrease with time in culture, are in keeping with previous literature, which has demonstrated that the distinct epigenetic patterns and enhanced phenotype in a given cell type‐derived iPSC are lost following repeat passaging 16, 17, 19, 21.

Throughout this study, few significant differences were found between the PHH‐ and HDF‐derived‐iPSCs. Recent work has suggested that the major determinant in HLC differentiation phenotype is the donor 22. This was found to be true for the gene expression analysis of the iPSC, definitive endoderm and EB samples. The gene expression profile of HLCs has been reported to be influenced by the genotype of the corresponding donor PHH 21, 35. Therefore, to reduce genotype‐derived donor variation and allow for a more representative comparison of the starting cell type, HLC samples were compared to the corresponding donor's PHH. Despite this analytical approach, no significant differences were found between PHH‐ and HDF‐derived HLCs.

While the donor appears to be a major determinant of phenotype, other nondefined factors, not related to starting cell type or donor, also appear to influence the hepatic differentiation capacity of iPSCs. This inter‐clonal variation contributed to the lack of significant findings between starting cell types. For example, albumin secretion was found to be generally higher in PHH‐derived clones; however, when all lines were compared, no significant differences were seen between PHH‐ and HDF‐derived lines. This was in part due to the donor 1 HDF‐derived line, Liv4FA, which displayed much greater albumin secretion and gene expression than all other lines. If Liv4FA is not included in the analysis, there is significantly greater HLC albumin secretion in lines derived from PHH. Thus, our results suggest that both donor‐ and clone‐dependent variation has greater influence on HLC phenotype than the starting cell type. Further work is, therefore, required to define the differences between iPSC clones which infer the capacity for hepatic differentiation.

Previous work has described the reprogramming of human hepatocytes as being relatively inefficient compared to HDFs and that it was only achievable with cells derived from relatively young donors 27. In contrast, we were able to generate clones from donors which were comparatively old (63 and 66 years old). Interestingly, despite using only four clones for the analysis, we were able to generate 10 clones from donor 3 (27 years old). These data point toward an enhancement of hepatocyte reprogramming efficiency in younger donors. The effect of age has been discussed in depth in other starting cell types; with multiple studies in mice showing a decline in reprogramming efficiency with age 36. However, the effect of age on reprogramming efficiency in human cells is more difficult to assess due to the unknown influence of the donor's genetic background. As a consequence, the effect of age is under debate; Sommer et al. 37, reported no significant correlation between age and efficiency, whereas, Trokovic et al. 38, did find a correlation, in a P21‐dependent manner.

The isolation of PHH is relatively well‐established; however, the lack of a bona fide cell surface marker and the sensitivity of the cells during the isolation process mean that, to the best of our knowledge, a 100% homogenous population of hepatocytes is not currently achievable. Therefore, there remains a possibility that not all of the iPSC clones derived from the PHH‐enriched population were derived from hepatocytes. However, if PHH‐derived clones were to be used in future experiments, the inability to guarantee a homogenous starting population of hepatocytes will likely remain; therefore, this work is also representative of the practical limitations associated with PHH as a starting cell source.

It must also be acknowledged that the slight enhancements seen in PHH‐derived clones may well be limited by the restricted differentiation maturity which is currently achievable in simple two‐dimensional HLC culture systems, as used in this study. In line with this, our PHH‐derived iPSCs do appear to show a significant trend (Supporting Information Fig. S6b) toward enhancement of phenotype at the hepatic endoderm stage which is not carried through to our HLC analysis. Previous work in mice has shown that the greatest advantage in HLC differentiation was achieved by reprogramming hepatoblasts, rather than adult hepatocytes 19. This may be a consequence of the relatively immature phenotype of HLCs. Therefore, future investigations into the starting cell type would require the development of protocols which produce a more mature phenotype; however, this in itself remains a major bottleneck in the field of HLCs 7. Moreover, through the inclusion of spontaneous differentiation assays and methylation analysis alongside our HLC comparisons, we have demonstrated that the starting cell type is unlikely to be of significant influence, even when protocols which allow for enhanced differentiation are developed.

## Conclusion

Taken together, the lack of any major advantage to PHH‐derived iPSCs is of significance to the field of iPSC‐derived HLCs, suggesting that any trend toward enhanced expression in PHH‐derived clones is not greater than the variation derived from the donor and other nondefined influencing factors. Furthermore, the access and phenotypic range of available PHH samples is very restricted in comparison to the easily accessible skin or blood samples used in the majority of current studies 24, 39. Therefore, the similarity between the cell types for all tested parameters is reassuring for current and future studies, particularly those which require specific genotypic backgrounds for disease studies, or a large phenotypic range for population‐representative research.

## Author Contributions

J.A.H.: conception and design, collection and/or assembly of data, data analysis and interpretation, manuscript writing; R.K.: conception and design, collection and/or assembly of data, data analysis and interpretation; C.S.P. and T.L.: collection and/or assembly of data, data analysis and interpretation; R.L.S.‐Y.: collection and/or assembly of data; M.E. and S.W.F.: provision of study material or patients; J.S.M. and N.R.K.: conception and design, administrative support; C.E.G.: conception and design, administrative support, manuscript writing; B.K.P.: final approval of manuscript.

## Disclosure of Potential Conflicts of Interest

J.M. is an employee of AstraZeneca and holds shares in the company. The other authors indicated no potential conflicts of interest.

## Supporting information

Supporting Information

Supporting Information

Supporting Information

Supporting Information

Supporting Information

Supporting Information

Supporting Information

Supporting Information

Supporting Information

Supporting Information
