# Supplementary figures and images for: Donor‐Dependent and Other Nondefined Factors Have Greater Influence on the Hepatic Phenotype Than the Starting Cell Type in Induced Pluripotent Stem Cell Derived Hepatocyte‐Like Cells
Source: Stem Cells Transl Med. 2017 Apr 29;6(5):1321–31. doi: 10.1002/sctm.16-0029 (PMC5442714; doi:10.1002/sctm.16-0029)

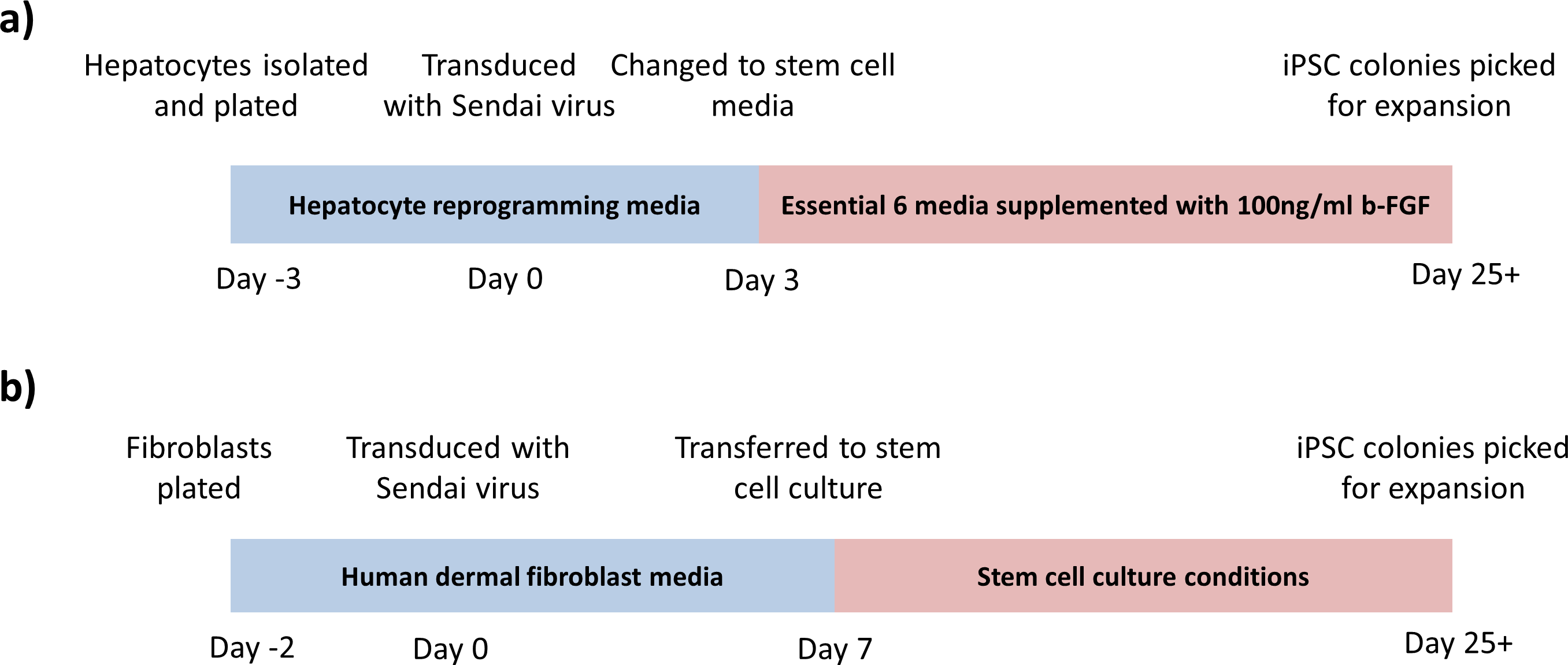

Supplement: Supplementary file 1 — Supporting Information [file SCT3-6-1321-s001.TIF]

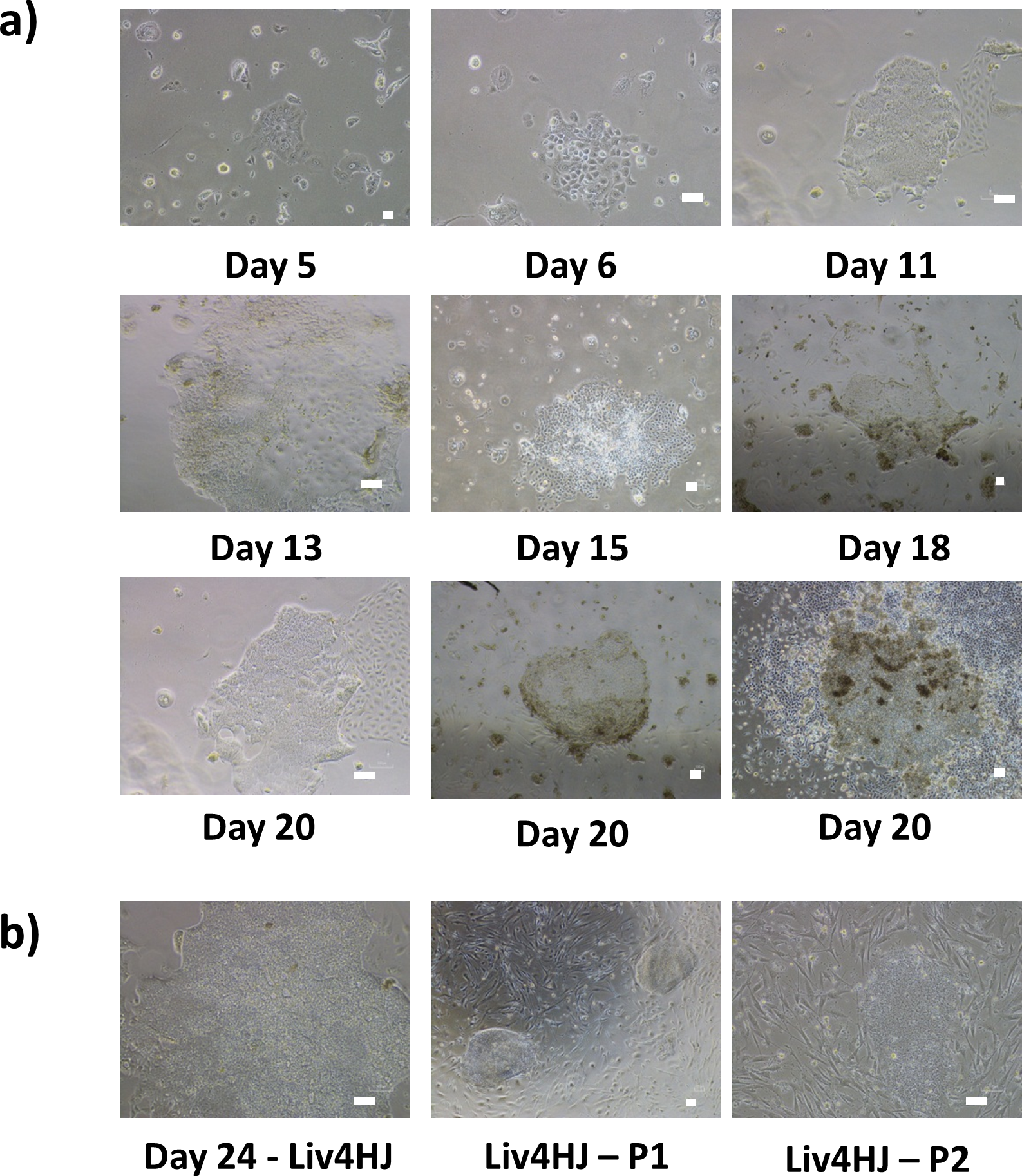

Supplement: Supplementary file 2 — Supporting Information [file SCT3-6-1321-s002.TIF]

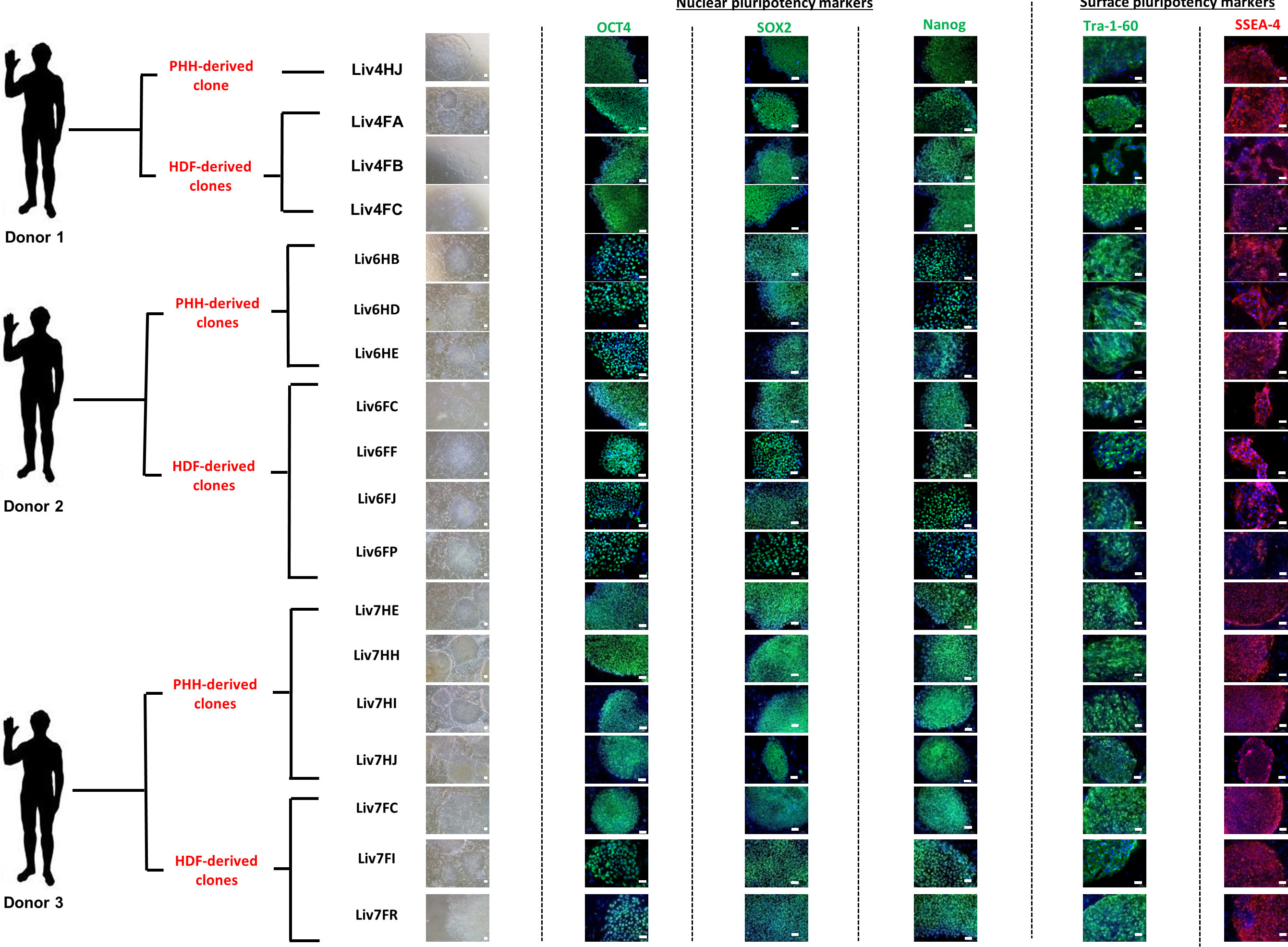

Supplement: Supplementary file 3 — Supporting Information [file SCT3-6-1321-s003.TIF]

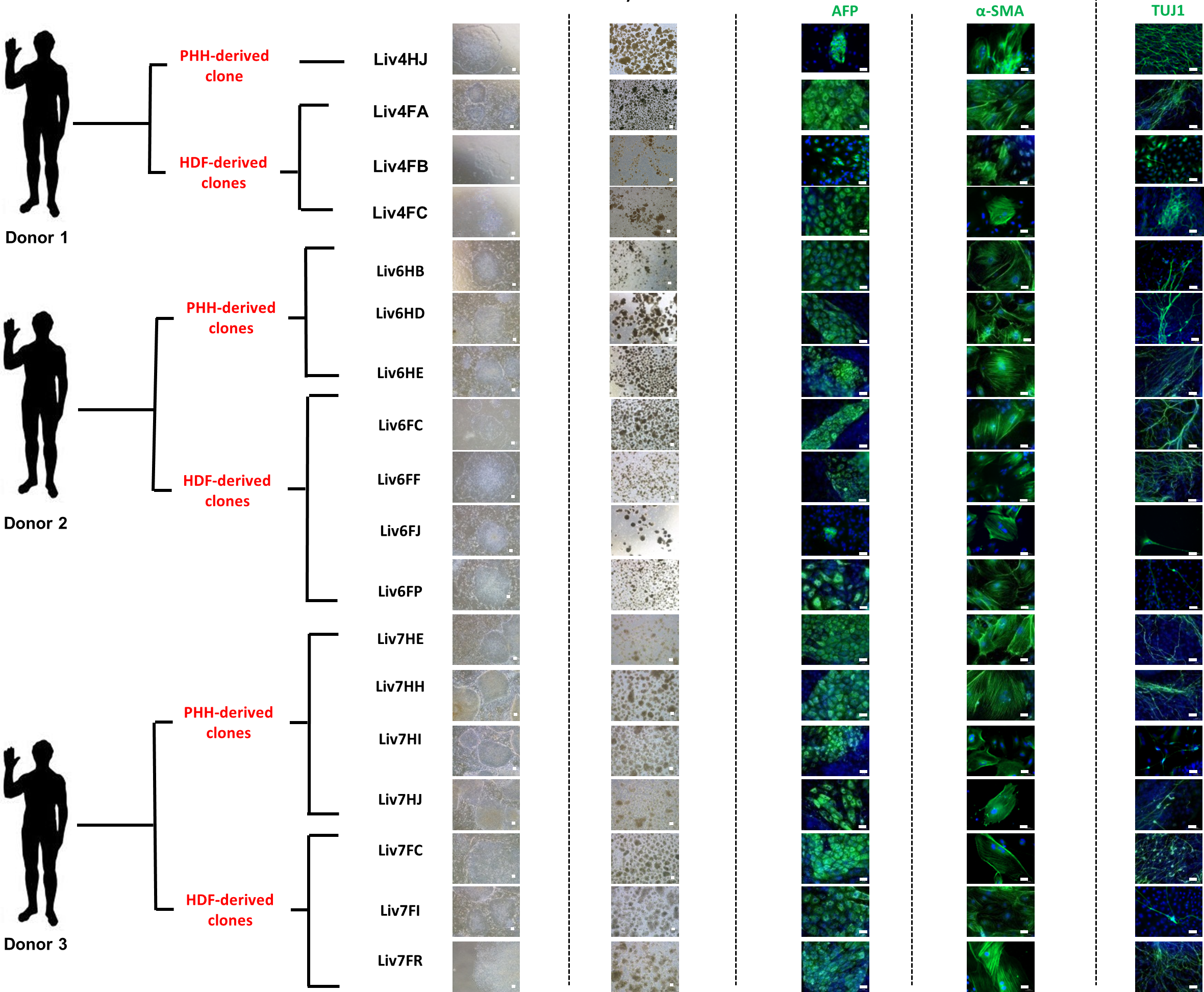

Supplement: Supplementary file 4 — Supporting Information [file SCT3-6-1321-s004.TIF]

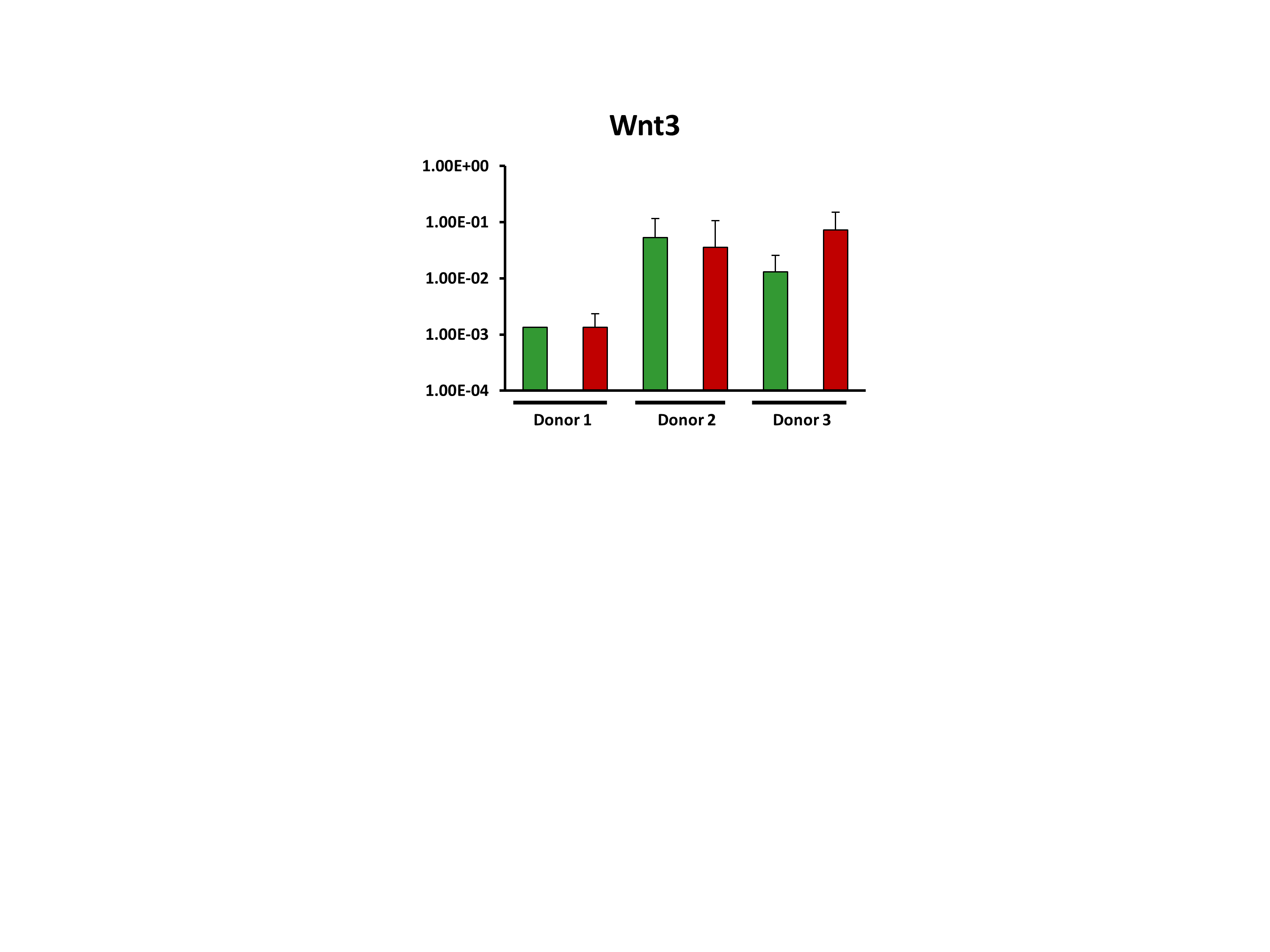

Supplement: Supplementary file 5 — Supporting Information [file SCT3-6-1321-s005.tif]

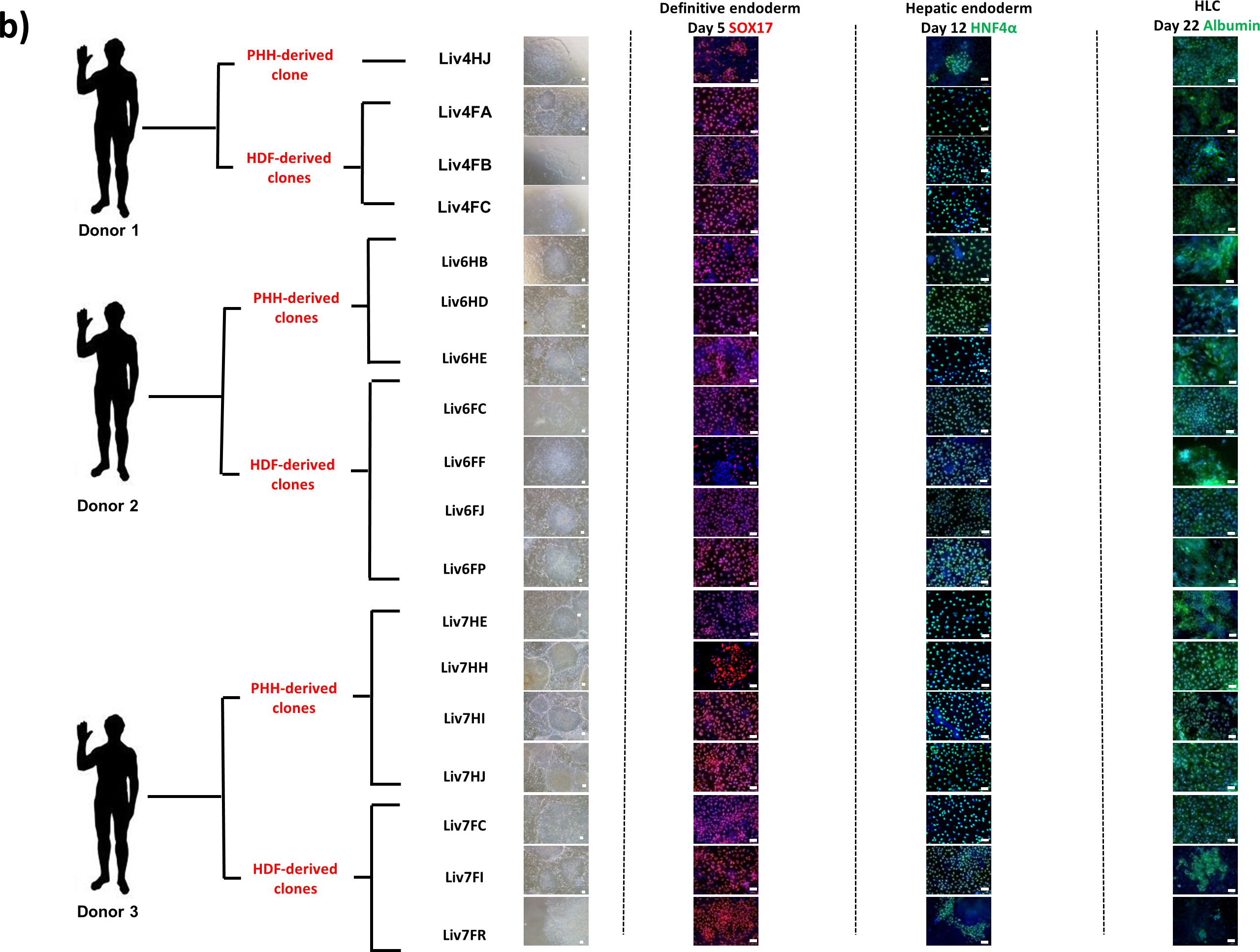

Supplement: Supplementary file 6 — Supporting Information [file SCT3-6-1321-s006.TIF]

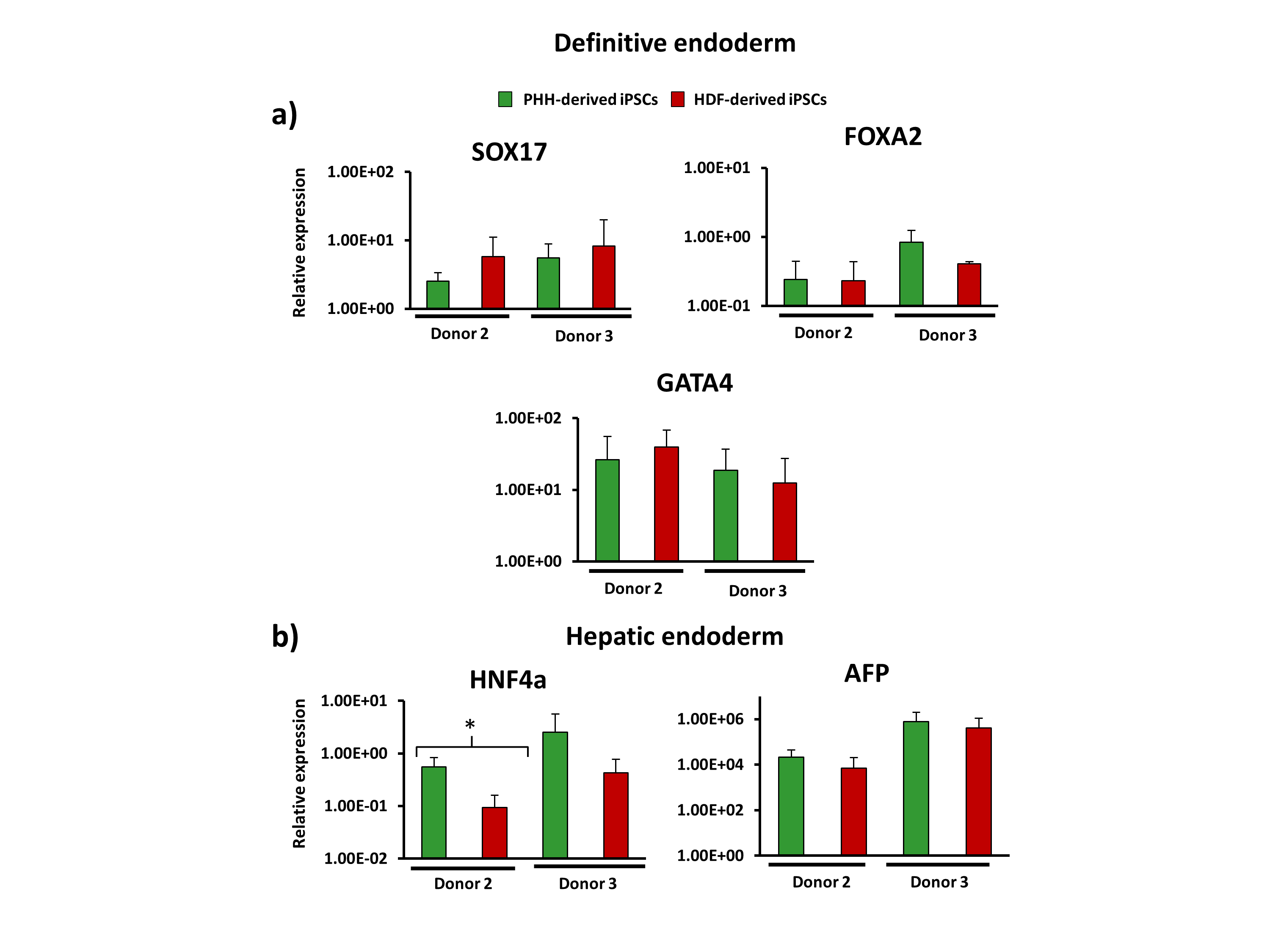

Supplement: Supplementary file 7 — Supporting Information [file SCT3-6-1321-s007.tif]

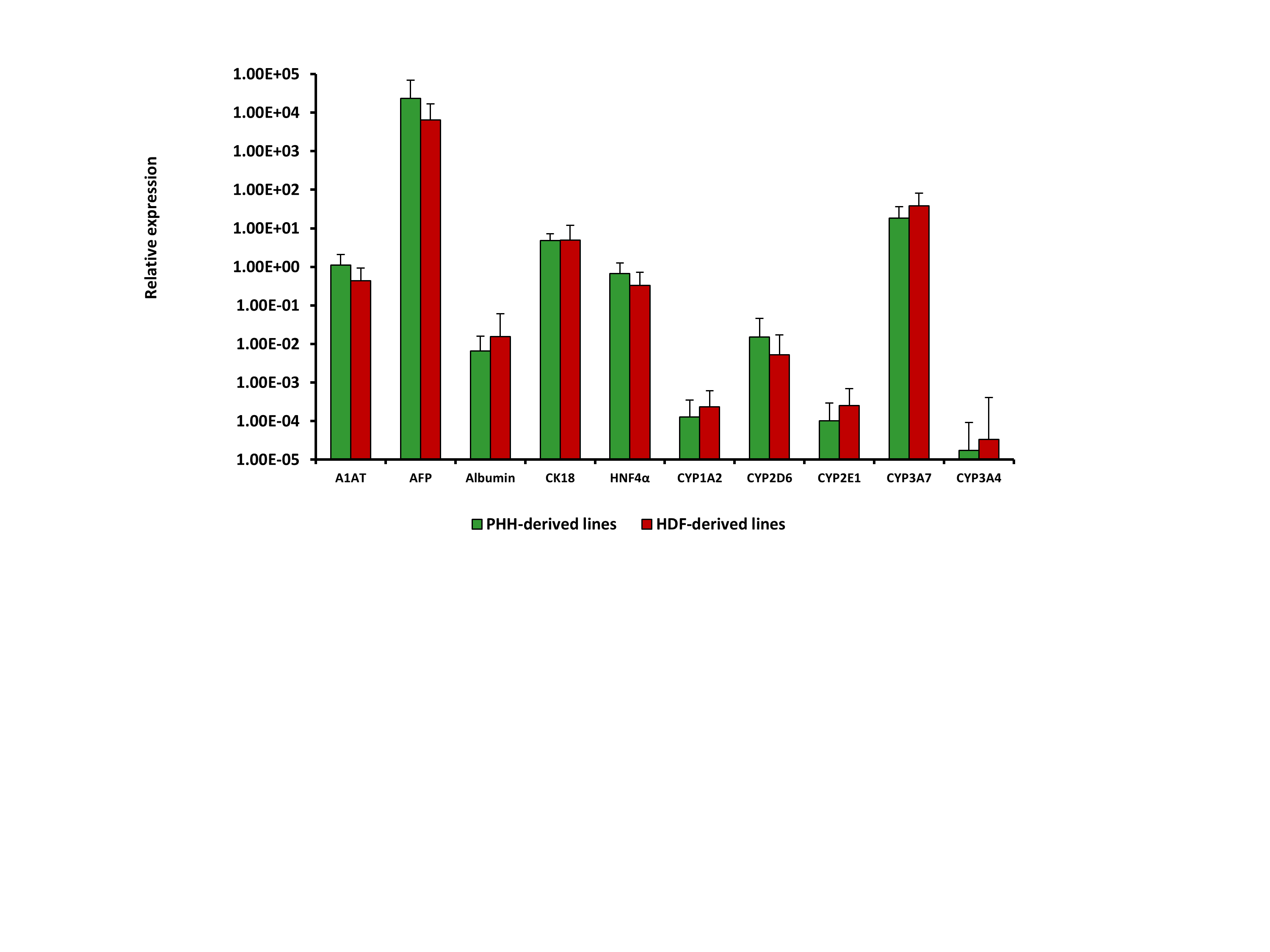

Supplement: Supplementary file 8 — Supporting Information [file SCT3-6-1321-s008.tif]
