# Supplementary material for: Donor‐Dependent and Other Nondefined Factors Have Greater Influence on the Hepatic Phenotype Than the Starting Cell Type in Induced Pluripotent Stem Cell Derived Hepatocyte‐Like Cells
Source: Stem Cells Transl Med. 2017 Apr 29;6(5):1321–31. doi: 10.1002/sctm.16-0029 (PMC5442714; doi:10.1002/sctm.16-0029)
Supplement: Supplementary file 9 — Supporting Information [file SCT3-6-1321-s009.docx]

**Supplementary figure legends**

**Figure S1:** Protocols for the reprogramming of somatic cells. a) PHH and b) HDF reprogramming protocols. Schematic diagram of the protocols used to reprogram each cell type. PHH: Primary human hepatocytes; HDFs: Human dermal fibroblasts; b-FGF: basic fibroblast growth factor

**Figure S2:** Reprogramming of PHH to IPSCs. **a)** The morphological changes during reprogramming and the different cell types which are generated during this period. **b)** Successfully generated iPSC clone with correct morphology and growth characteristics before and after manual picking and passaging. Magnification: x100, scale bar: 100µm; x40, scale bar: 100µm.

**Figure S3:** Generation and characterization of each iPSC line confirmed by morphology and immunofluorescence. Morphology of each line is shown next to the immunofluorescence images of nuclear pluripotency markers OCT4, SOX2 and Nanog and surface markers Tra-1-60 and SSEA-4. Immunofluorescence images: x200 magnification, 50µm scale bar. Light microscopy images: x100 magnification, 100µm scale bar.

**Figure S4:** Functional pluripotency characterization using embryoid body assay confirmed by immunofluorescence**.** Embryoid bodies generated from each iPSC line and stained for markers of the three germ layers associated with pluripotency: Endoderm (AFP), Mesoderm (α-SMA) and Ectoderm (Tuj1). Immunofluorescence images: x200 magnification, 50µm scale bar. Light microscopy images: x100 magnification, 100µm scale bar.

**Figure S5:** Wnt3 gene expression determined by q-PCR and presented as 2^-ΔΔ^CT relative to hESC comparator and normalized with GAPDH. Error bars represent standard deviation between the means of each PHH-/HDF-derived iPSC line which were derived from three individual differentiation cultures. Each sample tested by qRT-PCR was loaded in duplicate. (*) denotes p>0.05 unpaired T test.

**Figure S6:** Differentiation of all lines to hepatocyte-like cells and confirmation by immunofluorescence. **a)** A schematic diagram of the protocol used for the differentiation experiments, including the growth factors/small molecules used at each stage of differentiation. **b)** Immunofluorescence analysis of iPSCs during each stage of differentiation: Definitive endoderm (Sox17), Hepatic endoderm (HNF4α) and hepatocyte-like cells (albumin). Immunofluorescence images: x200 magnification, 50µm scale bar. Light microscopy images: x100 magnification, 100µm scale bar.

**Figure S7:** Comparison of gene expression during hepatocyte-like cell differentiation measured by qRT-PCR. **a)** Definitive endoderm associated genes at day 5 of differentiation. **b)** Hepatic endoderm associated genes at day 12 of differentiation. All genes shown as 2^-ΔΔ^CT relative to a hESC-derived control and normalized using GAPDH and Succinate dehydrogenase gene expression. Error bars represent standard deviation between the means of each PHH-/HDF-derived iPSC line which were derived from three individual differentiation cultures. Each sample tested by qRT-PCR was loaded in duplicate. N.B. donor 1 D.E and H.E samples were not generated during the low passage differentiation experiments and therefore are not included in this analysis. (*) denotes p>0.05 unpaired T test.

**Figure S8:** Combined gene expression of hepatocyte-like cells derived from PHH and HDF-derived lines. Gene expression of key hepatic genes obtained by q-PCR analysis and displayed as 2^-ΔΔ^CT relative to PHH of the corresponding donor. N.B. CYP3A4 was not detected in donor 1 and 2 HLCs, results shown are derived from donor 3 only. Results normalized using GAPDH and Succinate dehydrogenase. Error bars represent standard deviation between the means of each PHH-/HDF-derived iPSC line which were derived from three individual differentiation cultures. Each sample tested by qRT-PCR was loaded in duplicate. (*) denotes p>0.05 unpaired T test.
