# Supplementary material for: Donor‐Dependent and Other Nondefined Factors Have Greater Influence on the Hepatic Phenotype Than the Starting Cell Type in Induced Pluripotent Stem Cell Derived Hepatocyte‐Like Cells
Source: Stem Cells Transl Med. 2017 Apr 29;6(5):1321–31. doi: 10.1002/sctm.16-0029 (PMC5442714; doi:10.1002/sctm.16-0029)
Supplement: Supplementary file 10 — Supporting Information [file SCT3-6-1321-s010.docx]

| **Authors** | **Species** | **Starting cell types** | **Assays used** | **Improved phenotype?** |
| --- | --- | --- | --- | --- |
| (Liu et al., 2011) | Human | Hepatocytes  Fibroblasts  Keratinocytes  BM-MSCs | Albumin secretion  CYP3A4 activity | Yes, but non-significant |
| (Lee et al., 2012) | Mouse | Mouse ESCs  Fibroblasts  Hepatoblasts  Hepatocytes | Albumin secretion  HLC gene expression | Yes, but transient |
| (Kajiwara et al., 2012) | Human | PBMCs  Fibroblasts | Albumin/Urea secretion | No, donor dependent |
| (Takayama et al., 2014) | Human | Hepatocytes  Fibroblasts  PBMCs  HUVECs | Albumin secretion  CYP3A4 activity  TAT expression | Yes, but transient |

**Table S1**: Summary of the previously published investigations of starting cell type for hepatocyte-like cell differentiation

|  | Name | Sequence 5’-3’ | Modification |
| --- | --- | --- | --- |
| 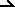 | HNF4ameth-F | TGGGTGATTAGAAGAATTAATAAG |  |
| 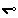 | HNF4ameth-Rb | CAACACAACCACCAAAAAC | 5’ biotin |
| 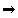 | HNF4ameth-S | TGATTAGAAGAATTAATAAGATA |  |
| 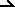 | FOXA2meth-F | TGTGATTGAAAAGTAATTTTGAA |  |
| 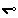 | FOXA2meth-Rb | CAAACAACCCCTCTAACAAC | 5’ biotin |
| 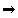 | FOXA2meth-S | GAAAAGTAATTTTGAAATA |  |

**Table S2**: Primer sequences used for pyrosequencing analysis.


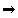
: Sequencing primer;
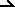
: Forward primer;
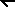
: Reverse primer;
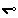
/
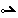
: Biotinylated primer.

| Antibody | Company | Dilution | Secondary | Dilution |
| --- | --- | --- | --- | --- |
| Oct4 | Abcam | 1:100 | Anti-rabbit alexafluor 488 | 1:750 |
| Sox2 | Abcam | 1:100 |  |  |
| Nanog | Abcam | 1:100 |  |  |
| AFP | Dako | 1:100 |  |  |
| α-SMA | Abcam | 1:100 |  |  |
| TUJ1 | Abcam | 1:500 |  |  |
| HNF4α | Santa-Cruz | 1:50 |  |  |
| Albumin | Abcam | 1:20 |  |  |
| Sox17 | RnD systems | 1:20 | Anti-goat alexafluor 568 | 1:750 |
| Tra-1-60 | BD Gentest | 1:10 | Conjugated 488 | N/A |
| SSEA-4 | BD Gentest | 1:10 | Conjugated 568 |  |

**Table S3:** Antibodies and related dilutions used for immunofluorescence-based characterization of the iPSC lines generated

| **Primer** | | **Sequence (5’ to 3’)** |
| --- | --- | --- |
| ALB | F | CCTGTTGCCAAAGCTCGATG |
|  | R | ATCTCCATGGCAGCATTCGC |
| A1AT | F | TCCGATAACTGGGGTGACCT |
|  | R | AGACGGCATTGTCGATTCACT |
| CYP1A2 | F | AGCACCTGCCTCTACAGTTGG |
|  | R | TGGTGGACTTTTCAGGCCTTT |
| CYP3A4 | F | TGTGCCTGAGAACACCAGAG |
|  | R | GTGGTGGAAATAGTCCCGTG |
| CYP3A7 | F | AGACGGGCTTCATCCAATGTG |
|  | R | ATGGTGCTAACTGGGGGTGG |
| CYP2E1 | F | ACCCTGAGATCGAAGAGAAGC |
|  | R | AAATGGTGTCTCGGGTTGCT |
| CYP2D6 | F | TTCCCAAGGGGTGTTCCTGG |
|  | R | TCACGGCTTTGTCCAAGAGA |
| CK18 | F | ACATCCGGGCCCAATATGAC |
|  | R | GGTGCTCTCCTCAATCTGCT |
| GAPDH | F | CTATAAATTGAGCCCGCAGCC |
|  | R | GCCCAATACGACCAAATCCGT |
| HNF4α | F | GTTGACGATGGGCAATGACAC |
|  | R | TCTTTGTCCACCACGCACTG |
| AFP | F | GCGGCCTCTTCCAGAAACTA |
|  | R | AATAATGTCAGCCGCTCCCT |
| SOX17 | F | GGATACGCCAGTGACGACCA |
|  | R | GACTTGCCCAGCATCTTGCTC |
| FOXA2 | F | ATTGCTGGTCGTTTGTTGAGG |
|  | R | TTCATGCCGTTCATCCCCAG |
| GATA4 | F | CGACACCCCAATCTCGATATG |
|  | R | GTTGCACAGATAGTGACCCGT |
| CXCR4 | F | GAAACCCTCAGCGTCTCAGT |
|  | R | AGTAGTGGGCTAAGGGCACA |
| WNT3 | F | GAGCCCAGAGATGTGTACTGC |
|  | R | CTTCTAATGGAGCCCCACCT |
| PDX1 | F | CAAAGCTCACGCGTGGAAAG |
|  | R | TTTTTCCACTTCATGCGGCG |
| ISL1 (Insulin) | F | ACAAGCAGCCGGAGAAGAC |
|  | R | TGGATATTAGTTTTGTCATTGGGCT |
| SDHA | F | TGGTTGTCTTTGGTCGGG |
|  | R | GCGTTTGGTTTAATTGGAGGG |
| OCT4 | F | AGACCATCTGCCGCTTTGAG |
|  | R | GCAAGGGCCGCAGCTT |
| SOX2 | F | AACCAGCGCATGGACAGTTAC |
|  | R | TGGTCCTGCATCATGCTGTAG |
| NANOG | F | GGCTCTGTTTTGCTATATCCCCTAA |
|  | R | CATTACGATGCAGCAAATACGAGA |

**Table S4:** Primer sequences used for qRT-PCR analysis.

|  | **DONOR 1** | **DONOR 2** | **DONOR 3** |
| --- | --- | --- | --- |
| **Age at surgery** | 66 | 63 | 27 |
|  |  |  |  |
| **BMI** | 27.9 | 31.2 | 32.1 |
|  |  |  |  |
| **Sex** | Male | Female | Male |
|  |  |  |  |
| **Diagnosis** | Colorectal carcinoma  with liver metastases | Colorectal carcinoma  with liver metastases | Focal nodular hyperplasia |
|  |  |  |  |
| **Co-morbidity** | Type II diabetes mellitus | Hypertension  Chronic liver disease  Type II diabetes mellitus | None |
| **No. of PHH-derived clones** | 1 | 5 | 10 |

**Table S5:** Details of the donors from which the cells used for reprogramming were derived, including key parameters such as age, co-morbidities and the number of iPSC clones derived from each donor PHH.
